# Characterization of Metronidazole-Resistant *Giardia intestinalis* Lines by Comparative Transcriptomics and Proteomics

**DOI:** 10.3389/fmicb.2022.834008

**Published:** 2022-02-10

**Authors:** Sascha Krakovka, Ulf Ribacke, Yukiko Miyamoto, Lars Eckmann, Staffan Svärd

**Affiliations:** ^1^Department of Cell and Molecular Biology, Biomedical Center (BMC), Uppsala University, Uppsala, Sweden; ^2^Department of Microbiology, Tumor and Cell Biology (MTC), Karolinska Institutet, Stockholm, Sweden; ^3^Department of Medicine, University of California, San Diego, La Jolla, CA, United States; ^4^SciLifeLab, Uppsala University, Uppsala, Sweden

**Keywords:** diarrhea, antibiotic resistance, RNAseq, proteomics, small intestine, protozoa

## Abstract

Metronidazole (MTZ) is a clinically important antimicrobial agent that is active against both bacterial and protozoan organisms. MTZ has been used extensively for more than 60 years and until now resistance has been rare. However, a recent and dramatic increase in the number of MTZ resistant bacteria and protozoa is of great concern since there are few alternative drugs with a similarly broad activity spectrum. To identify key factors and mechanisms underlying MTZ resistance, we utilized the protozoan parasite *Giardia intestinalis*, which is commonly treated with MTZ. We characterized two *in vitro* selected, metronidazole resistant parasite lines, as well as one revertant, by analyzing fitness aspects associated with increased drug resistance and transcriptomes and proteomes. We also conducted a meta-analysis using already existing data from additional resistant *G. intestinalis* isolates. The combined data suggest that *in vitro* generated MTZ resistance has a substantial fitness cost to the parasite, which may partly explain why resistance is not widespread despite decades of heavy use. Mechanistically, MTZ resistance in *Giardia* is multifactorial and associated with complex changes, yet a core set of pathways involving oxidoreductases, oxidative stress responses and DNA repair proteins, is central to MTZ resistance in both bacteria and protozoa.

## Introduction

Diarrheal disease is a major health threat for children under the age of five in developing countries, causing more than 715,000 deaths yearly (Kirk et al., 2015). It is the leading cause of malnutrition in this age group, which in turn results in stunting and general developmental retardation (Kirk et al., 2015). A large proportion of the 1.7 billion annual cases of diarrhea could be prevented by providing clean drinking-water and better hygienic measures but as this is difficult to achieve in low resource settings effective treatment of infection remains vital. One of the most commonly used drugs to treat diarrheal and other infections is metronidazole (MTZ). This 5-nitroimidazole is popular because it combines several aspects that are beneficial: The drug is cheap and can be bought over-the-counter (World Health Organization [WHO], 2005) and it targets a broad spectrum of anaerobic pathogens, ranging from bacteria to eukaryotic parasites (Leitsch, 2019). These aspects combined have led to the inclusion of MTZ on the WHO list of essential medicines (World Health Organization [WHO], 2019). In addition, reports of antimicrobial resistance toward MTZ were scarce for many years after its introduction (Cosar and Julou, 1959; The Nitroimidazole Family of Drugs, 1978). However, this has changed recently, with resistance rates reported to be on the rise for several pathogens where MTZ is the first line treatment (Thung et al., 2016; Carter et al., 2018).

Despite its long and widespread use, it is still not fully understood how MTZ exerts cytotoxicity on bacteria and protozoa, since numerous target molecules have been identified but their relative importance for cell killing remains unclear (Lauwaet et al., 2020). The drug is assumed to enter cells via passive diffusion, although recent studies suggest involvement of active mechanisms (Dingsdag and Hunter, 2018). MTZ is a prodrug and needs to be activated by partial reduction upon cell entry. This can either be catalyzed by oxidoreductases of various classes, like pyruvate ferredoxin oxidoreductases (PFORs) and nitroreductases and/or by reducing agents (Dingsdag and Hunter, 2018). In support of this, oxidoreductases are often mutated or differentially expressed (DE) in MTZ resistant pathogens (Gerrits et al., 2004; Chong et al., 2014; Moura et al., 2014; Lauener et al., 2019). The exact structure of the active molecule of MTZ is unknown but a toxic radical is assumed to play a role as hinted at by the common up-regulation of general repair mechanisms, oxidative stress responses and a halt of DNA synthesis. The exact site of damage has similarly not been established but effects are often seen at the DNA level (Dingsdag and Hunter, 2018). The active form of MTZ is rapidly converted back to the prodrug in the presence of oxygen, which explains the selectivity of this drug for anaerobic or microaerophilic cells (Perez-Reyes et al., 1980).

Most organisms commonly treated with MTZ can develop resistance in the clinical setting (Leitsch, 2019). Treatment efficacy in general is influenced by environmental oxygen levels and the availability of antioxidants like cysteine (Milne et al., 1978; Leitsch, 2017). Aside from this, several pathogen-specific resistance mechanisms have been proposed. *Trichomonas vaginalis* was reported to have an elevated intracellular oxygen content due to downregulated oxygen-scavenging, which has been called the aerobic resistance type (Meingassner et al., 1978). Furthermore, in many MTZ resistant *T. vaginalis* strains, PFOR and related pathways are lost but this is not sufficient to generate high level of resistance by itself (Muller and Gorrell, 1983; Leitsch et al., 2009). Another pathogen showing an aerobic resistance type is the gram-positive bacterium, *Clostridioides difficile*. Lower concentrations of MTZ promote biofilm formation and reduced MTZ sensitivity; furthermore oxidoreductases have been reported to be differentially expressed in resistant clones (Chong et al., 2014; Moura et al., 2014; Vuotto et al., 2016). Another type of resistance mechanism is found in gram-negative bacteria from *Bacteroides spp*. In these bacteria strains with MTZ resistance have been reported to rely on drug efflux pumps and a class of genes called NIM (nitroimidazole resistance gene) (Husain et al., 2013). These genes are suggested to catalyze the inactivation of MTZ, although they appear not to be sufficient for high level resistance phenotypes (Pumbwe et al., 2007; Leitsch et al., 2014b). In *Helicobacter pylori*, several pathways appear to be involved in resistance, including the oxidative stress response and two nitroreductases, RdxA, an oxygen-insensitive nitroreductase with FMN (Flavin mononucleotide) and NADH (Nicotinamide adenine dinucleotide) as cofactors as well as FrxA, a less characterized nitroreductase (Chua et al., 2019; Lauener et al., 2019). Another example is the intestinal protozoan parasite *Giardia intestinalis* in which MTZ resistance levels rose from 15 to 40% in just five years in London in the last decade (Carter et al., 2018).

*Giardia intestinalis* is a unicellular pathogen which causes some 190 million symptomatic diarrhea cases and loss of 171,100 DALYS (daily adjusted life years) every year (Kirk et al., 2015). Giardiasis has a broad range of symptoms, varying from asymptomatic to severe abdominal cramping and “explosive” diarrhea. In most patients, the infection is cleared by the immune system within two weeks, however, in some, infections can become chronic. In a fraction of all patients - both chronically infected and patients that clear the infection - long-term effects like irritable bowel syndrome (IBS), chronic fatigue syndrome and food allergies develop which can persist for over a decade. In young and malnourished patients, severe diarrhea can exacerbate malnutrition, hinder development, and even cause death (Bartelt and Sartor, 2015; Einarsson et al., 2016a).

Metronidazole is the standard treatment in giardiasis, since other available treatments options are either more expensive, less efficient or have more severe adverse effects (Gardner and Hill, 2001; World Health Organization [WHO], 2005; Carter et al., 2018; Argüello-García et al., 2020). Most of our knowledge of MTZ resistance in *G. intestinalis* has been generated using laboratory generated strains, since no clinically resistant *G. intestinalis* strains have been axenized and characterized in detail on the molecular level (Lemée et al., 2000; Tejman-Yarden et al., 2011). No major genomic changes have so far been identified in MTZ resistant *G. intestinalis* isolates, unlike what is commonly seen in bacteria (Ansell et al., 2017; Saghaug et al., 2019). The low level of genetic fixation of MTZ resistance is underlined by the fact that resistant *G. intestinalis* strains can revert to sensitive both after en- and excystation and after growing for several generations without selective pressure (Tejman-Yarden et al., 2011). On the other hand, increasing levels of clinically MTZ resistant *G. intestinalis* are transferred between patients, implying that resistance is a stable, transmissible phenotype (Requena-Méndez et al., 2014; Carter et al., 2018). Very few studies have so far investigated *in vitro* generated MTZ resistant *G. intestinalis* strains at the molecular level and all strains have been limited to assemblage AI (Ansell et al., 2017; Emery et al., 2018; Müller et al., 2019). The findings to date point toward large mechanistic differences between isolates, suggesting that MTZ resistance in *G. intestinalis* assemblage AI is not unimodal, but our current understanding is clearly incomplete.

Here, we aimed to expand our understanding of MTZ resistance in *G. intestinalis* through the analysis of two additional MTZ resistant assemblage AI lines, M1 and M2, as well as one revertant, M1NR, previously generated by Tejman-Yarden et al. (2011). We analyzed specific phenotypes and the total transcriptomes and proteomes, followed by comparisons with already existing data from other isolates. The combined data suggest that individual resistant lines employ unique combinations of a set of resistance mechanisms but also reveal a conservation of MTZ resistance mechanisms in bacteria and protozoa, with a special importance of certain oxidoreductases and changes in intracellular levels of oxygen in most cases.

## Materials and Methods

### Cell Culture

Four isogenic *G. intestinalis* isolates were used in this study: WB (ATCC 50803); clone C6/A11 as well as metronidazole resistant strains (WB-) M1 (WBC6/A11-M1), (WB-) M2 (WBC6/A11-M2) and revertant (WB-) M1NR (WBC6/A11-M1NR). The A11 starting clone was generated by limited dilution of WB C6 in order to reduce potential sub-clonal genetic diversity (Birkeland et al., 2010). The resistant strains and the revertant were described by Tejman-Yarden et al. (2011). Lines were maintained in TYI-S-33 medium according to Keister (1983). For the MTZ resistant strains the medium was supplemented with 10 μM MTZ and the MTZ stress was relieved for the last passage approximately 20 hours before experiments.

### Establishment of EC_50_ and EC_90_ Values

All four lines were evaluated for their susceptibility to MTZ as described before (Tejman-Yarden et al., 2011). In short, culture tubes containing parasites were chilled on ice for 15 min, agitated repeatedly to ensure detachment, and cells were enumerated in a hematocytometer. Cells were diluted to 1 × 10^4^ cells/mL for WB and M1NR and 2 × 10^4^ cells/mL for M1 and M2 to adjust for previously reported differences in growth. 40 μL of these preparations were seeded into 96-well plates and incubated at 37°C for 2 h to allow for cell recovery under anaerobic conditions. MTZ was diluted in TYI-S-33 immediately before experiments and added to wells at final concentrations ranging from 1 to 200 μM (1, 2, 5, 10, 20, 50, 100, and 200 μM). For all drug concentrations, DMSO controls were included to adjust for solvent effects. After 48 h incubation at 37°C under anaerobic conditions, CellTiter-Glo reagent (Promega) was added and the solutions were mixed by shaking for 10 min. A Tecan plate reader (Infinite M200 Pro) was used to measure luminescence after allowing the mix to settle for 10 min. Based on the survival rates at each concentration, a logistic curve was fitted to the results from ten biological replicates per strain, and EC_50_ and EC_90_ values were calculated from the equation describing the respective curve. The equations had the general form:

$$y=a+\frac{\left(b-a\right)}{\left(1+{\left(\frac{x}{c}\right)}^{d}\right)}$$

with a, minimum survival rate; b, maximum survival rate; c, estimated EC_50_; d, Hill coefficient; y, percentage surviving cells; ×, concentration of MTZ. The equations were solved with the R onboard non-linear least squares method.

### Growth Rate Calculations

The same experiments that were used to determine EC_50_ values for MTZ was used to calculate growth rates for untreated controls. A linear correlation of parasite numbers to luminescence levels was established by measuring luminescence for different parasite numbers with CellTiter-Glo, which was added after two hours of attachment to the plates, and selecting the linear part of the curve (10^4^ to 5 × 10^5^ cells) for every line. An equation was fitted to this linear part and used to calculate cell numbers for all control wells. Averaged cell numbers for all lines were inserted into the formula $\frac{I\,n\,c\,u\,b\,a\,t\,i\,o\,n\,t\,i\,m\,e}{l\,o\,g_{2}\,\left(\frac{F\,i\,n\,a\,l\,c\,e\,l\,l\,c\,o\,u\,n\,t}{S\,t\,a\,r\,t\,c\,e\,l\,l\,c\,o\,u\,n\,t}\right)}$ to calculate doubling times. Significance was calculated with Welch’s *t*-test.

### Encystation Efficiency

**FIGURE 1:**
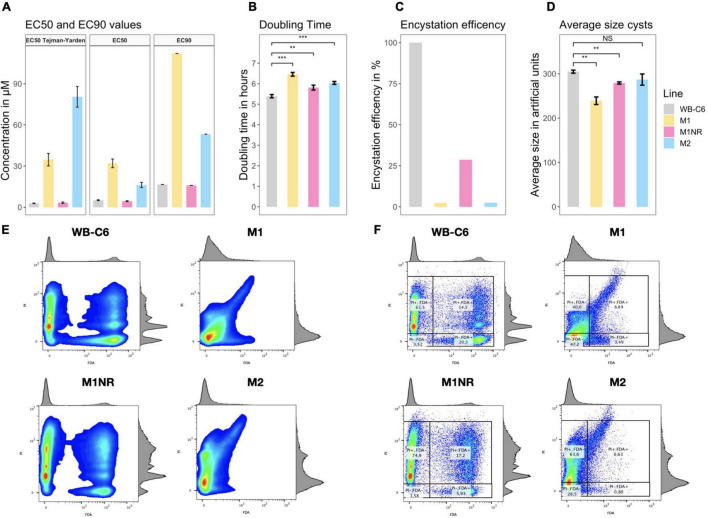
Overview of phenotypic data. **(A)** EC_50_ and EC_90_ values for the lines WB-C6, M1, M1NR, and M2 are presented. The first tile of the panel are the value from the original study by Tejman-Yarden et al. (2011) while the other two tiles are our data. Concentrations are given in μmol/l or μM. **(B)** The doubling time in hours for the same for lines is presented. **(C)** Encystation efficiency is given; WB-C6 is set to 100% and the viable cysts number for the other lines are compared to that as a percentage. **(D)** The mean values of the forward scattering channel for alive cysts are given separated by line. **(E,F)** diagrams of FDA (*X*-axis) and PI (*Y*-axis) fluorescence can be seen. Histograms of the respective populations are projected on the other side of the graph. Higher amounts of cell at any point of the graph are indicated by a color change from blue (lowest) to green to yellow to red (highest). **(F)** percentage are given for the subpopulations while those are omitted in panel **(E)** for better visibility. Significance is given when applicable as NS, non-significant, **p-value < 0.01, ***p-value < 0.001.

Encystation assays were performed in 10 mL flatside tubes in five biological replicates. Cell numbers were enumerated as described above and diluted to 2 × 10^5^ cells/mL in 10 ml fresh TYI-S-33. Cells were incubated 37°C for 3 h to allow for attachment before medium was changed to encystation medium according to the Uppsala encystation protocol (Einarsson et al., 2016b). After 48 h incubation at 37°C, cells were harvested and transferred to sterile H_2_O in which they were kept at 4°C for at least three days. Cysts were live/dead stained with fluorescein diacetate (FDA) and propidium iodide similar to what has been described for *Giardia muris* cysts (Schupp and Erlandsen, 1987). In short, FDA was stored as a stock solution of 25 mM in acetone and diluted on the experiment day to working solution by adding 40 μL to 10 mL PBS, pH = 6. Cysts were counted and around 10^6^ cells were stained by adding 100 μL FDA working solution and 40 μL of Propidium Iodide Ready Flow Reagent (Invitrogen), before adjusting the volume to 200 μL with PBS and incubating on ice for 20 min. Typical staining can be seen in Supplementary Figure 1. Cells were counted in a MACSQuant VYB cell counter using the blue laser (488 nm) and B1 filter (525 nm/50 nm) for FDA and the yellow laser (561 nm) and Y2 filter (615 nm/20 nm) for PI. 100 μl of every sample were evaluated for each analysis. Typical graphs of side and forward scattering as well as fluorescence for one sample from each line can be seen in Figures 1E,F and Supplementary Figures 2–4.

### Transcriptomics

Ten biological replicates of *G. intestinalis* lines WB, M1, M1NR and M2 were grown to around 80% confluence in parallel in TYI-S-33 in 15 mL Falcon tubes. Cells were harvested by chilling on ice for 10 min, agitated against a hard surface for detachment, and pelleted at 2000 xG for 5 min at 4°C. Cells were immediately lysed in 1 mL TRIzol (Thermo Fisher Scientific) and snap-frozen in liquid nitrogen. RNA was extracted according to the manufacturer’s instructions. The RNA pellet was resuspended in RNase-free H_2_O and treated with DNase (DNase I, Amplification Grade, Cat# 18068015, Thermo Fisher Scientific) in the presence of RiboLock RNase inhibitor (#EO0381, Thermo Fisher Scientific) and purified again by phenol-chloroform extraction with glycogen added in the ethanol precipitation step. The dried pellets were again resuspended in RNase-free H_2_O and yields were determined using a Qubit fluorometric quantification device from Thermo Fisher Scientific. All samples were also analyzed on Tris/Borate/EDTA gels containing SYBR safe (Cat# S103102, Thermo Fisher Scientific) and visualized on a UV illuminator to assess appropriate RNA integrity. Five replicates where randomly chosen for each strain and sent to SciLife-Lab in Uppsala, Sweden^1^ for analysis by RNA sequencing. Sequencing libraries were generated with the TruSeq stranded mRNA library preparation kit (Cat# 20020594/5, Illumina Inc.) including polyA selection. Sequencing was done on a NovaSeq with 150 bp paired end v1 sequencing chemistry. Raw data can be retrieved at GEO (Barrett et al., 2013) under accession number GSE139624^2^. FASTQ files were mapped with STAR v.2.5.4a to the current WB reference genome (Xu et al., 2020) and STAR counts were used for analysis. Gene differential expression analysis was carried out in R using the edgeR (Robinson et al., 2010) v3.26.8 workflow. Only genes with at least two million reads in 5 samples were included. Clustering of all samples after normalization can be found in Supplementary Figure 5. A quasi-likelihood *F* test was used to determine significant differential gene expression. Expression levels for all five replicates of each strain were compared to the replicates of wild-type WB and differential expression profiles were established, with a priori assumption of false discover rate (FDR) adjusted *p*-values < 0.05 for significance using the Benjamini-Hochberg method. All transcriptomic results can be found in Supplementary Table 2.

### Proteomics

For proteomics, four replicates of the WB, M1, M1NR, and M2 lines, established from the same starting isolate as for transcriptomics, were grown to 80% confluence in TYI-S-33 without MTZ. M1 was also grown in four replicates in TYI-S-33 with 20 μM MTZ. All samples were harvested as described above until the cells were pelleted. Cell pellets were washed three times in PBS and snap-frozen in liquid nitrogen and stored at −80°C until processed by the Uppsala University proteomics facility. The samples were lysed in 1% β-octyl glucopyranoside and 6 M urea containing lysis buffer, reduced, alkylated, and on-filter digested with trypsin. They were centrifuged to dryness before resuspended in 30 μL 0.1% formic acid and diluted further four times before mass spectrometry. Peptides were separated on a 150 min gradient in a C18-column (reverse-phase) and analyzed on a Q Exactive Plus Orbitrap mass spectrometer (Thermo Fisher Scientific). The mass spectrometry data have been deposited to the ProteomeXchange Consortium via the PRIDE (Perez-Riverol et al., 2019) partner repository with the dataset identifier PXD027813 and 10.6019/PXD027813. Analysis of the raw data was done with MetaMorpheus version 00.313 (Solntsev et al., 2018) using the following strategy: Search- calibration- post-translational modification discovery- search. The final search was done with following settings: Max missed cleavages: 2; Min peptide length: 7; one peptide was enough to identify a protein; matching between the two runs was used, the quantification results were normalized. 1% FDR was set as cut-off. The version 50 from GiardiaDB for strain WB was used as annotated protein file^3^. Result files for all samples containing LFQ values, unique peptide numbers and modifications for each protein found were used to carry out DE analysis and modification analysis in R. LFQ values were normalized using the normalization approach from edgeR to account for differences in total protein levels. Clustering of all samples before and after normalization can be found in Supplementary Figure 6. DE analysis was done using the DEqMS (Zhu et al., 2020) workflow for label free proteomics and a DEqMS *p*-value of 0.05 or less was deemed significant. Welch’s *t*-test values for comparison were calculated with the base R *t*-test function. All proteomic results can be found in Supplementary Table 3.

### Gene Enrichment Analysis

Lists of gene IDs matching the criteria in question were exported from R and used to perform GO-term enrichment analysis for molecular function in GiardiaDB using both curated and computed evidence. GO-terms with *p*-values smaller or equal to 0.01 were considered significant and used for further interpretations.

### Meta-Comparisons of Transcriptomic and Proteomic Datasets From Resistant Isolates

All comparisons were done in R version 4.0.4 (2020-02-15) – “Lost Library Book.” In short, data from transcriptomics and proteomics experiments were bundled into a single data frame and expression patterns of groups of genes implied by previous data on phenotypic behavior, genomics, transcriptomics, and proteomics of MTZ resistant *G. intestinalis* strains were examined. An open search for DE genes in all or several resistant lines was conducted as well. Data from Ansell et al. (2017) and Müller et al. (2019) were reanalyzed in the way described above and included in the analysis to strengthen the results. Data from Emery et al. (2018) was included into the analysis based on their results. For comparability with the transcriptomics datasets, proteomics data was transformed into log2 values. Resulting tables can be found in Supplementary Tables 2–11. Figures were created with the pheatmap and formattable packages. Scripts are available upon request.

## Results

### Metronidazole Resistance Has Multifactorial Fitness Costs in *Giardia intestinalis*

As MTZ resistance has previously been shown to be potentially reversible (Tejman-Yarden et al., 2011) and therefore possibly unstable, we deemed it essential to independently determine MTZ inhibitory concentrations for the M1, M2, M1NR, and WB-C6 lines of *G. intestinalis* to confirm the prior findings (Tejman-Yarden et al., 2011). Consistent with previous data, we found WB-C6 and the revertant M1NR to display very similar sensitivities to MTZ with average EC_50_ values of 5.3 and 4.8 μM and EC_90_ values of 16.8 and 16.0 μM, respectively (Figure 1A and Supplementary Table 1). Similarly, the M1 and M2 lines were confirmed to be resistant to MTZ, with at least threefold higher EC_50_ and EC_90_ values compared to the sensitive lines. Levels of MTZ susceptibility correlated well with the prior determinations, except for M2, for which the newly determined EC_50_ was roughly 20% of the originally reported EC_50_ (16.35 vs. 80.4 μM). For all lines, the EC_90_ values were between three and four times the respective EC_50_ values (Figure 1A).

To explore whether drug resistance had any associated fitness costs, we determined the doubling times for all lines. M1 and M2 were found to grow slower by 16 and 26%, respectively, when compared to both the parental line and the revertant M1NR (Figure 1B and Supplementary Table 1). Thus, slower growth and greater doubling times tracked well with increased resistance, indeed suggesting MTZ resistance imposes a disadvantage to the parasite under drug-free conditions. Tejman-Yarden et al. (2011) had previously analyzed the lines for differences in attachment efficiency and infectivity of suckling mice and gerbils and saw a similar pattern, with reduced fitness for both measures associated with elevated MTZ resistance. Infections were performed in the prior work with trophozoites but the infectious form of *Giardia* is the cyst, so we decided to study if cyst formation (encystation) is also affected by resistance. WB-C6 had by far the highest encystation efficiency *in vitro* and a very high viability of the generated cysts (Figures 1C,D and Supplementary Table 1). From 10^6^ WB-C6 trophozoites an average of 5.8 × 10^4^ viable cysts were generated (viable or complete cysts show no signal for the dead stain, PI and high signal for the live stain, FDA, see Supplementary Figure 1). The number of viable cysts from WB-C6 was set to 100% and was used to compare the encystation efficiencies of the other lines. For the revertant M1NR, we observed that the encystation efficiency was around one quarter of what was seen for WB-C6 (Figure 1C and Supplementary Table 1). For both the resistant lines, a reduced level of encystation was observed (∼2.5%, Figure 1C and Supplementary Table 1). Flow cytometry analyses also showed that M1 and M1NR cysts were markedly smaller than the WB-C6 and M2 cysts (Figure 1D, Supplementary Figure 2, and Supplementary Table 1).

Another interesting aspect was observed when comparing the signal distributions for the different lines in the PI channel (Figures 1E,F). Since the PI stain labels the permeable, incomplete cysts after water treatment and it binds to DNA it can be used to study the DNA content in the incomplete cysts. For WB-C6 and M1NR, different populations can be distinguished, which show different levels of PI accumulation. For M2, this can be seen to a limited extend as well, while M1 cells showed mostly a weak PI positive signal (Figures 1E,F). Because the signal strength of PI is correlated to DNA content in the cells, we used it to evaluate the cell ploidy. The findings in M1 cells suggest that the DNA replication step, occurring in the late part of encystation, is incomplete since there are very few cysts with fully replicated DNA (16N, Supplementary Figures 3, 4) (Bernander et al., 2001). In addition, while both WB-C6 and M1NR trophozoites stopped growing and decreased in numbers after induction of encystation, this was not the case for M1 and M2, as they continued to grow as trophozoites in the encystation medium, underlining that the cells had defects in the initiation of encystation. Thus, the encystation process is dramatically disturbed in the MTZ resistant lines. In conjunction with the observed slowing in growth and the prior reports of decreased attachment and reduced animal infectivity of trophozoites, it is clear that MTZ resistance imposes multifactorial fitness costs on the parasites.

### Transcriptomic Responses Associated With Metronidazole Resistance Are Heterogenous of Low Amplitude and Reversible

**FIGURE 2:**
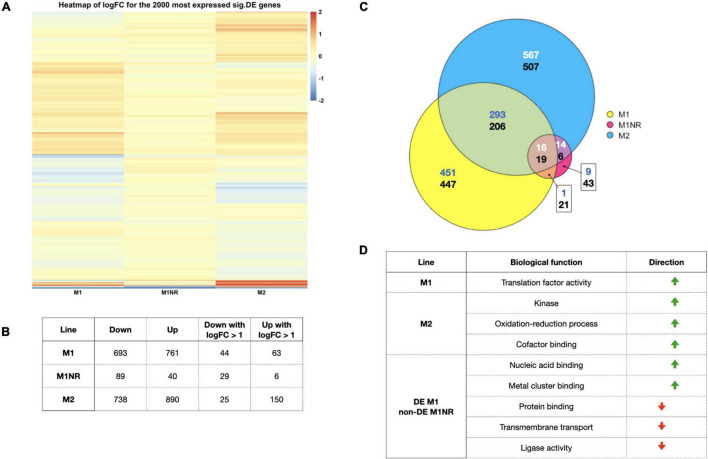
Overview of the data from our transcriptomics analysis. **(A)** A heatmap of the log2 fold changes for the top 2,000 expressed significantly DEG based on CPM values for the comparisons of M1, M2, and M1NR with WB is shown. A cut-off of 2 was used to enhance visualization of smaller fold changes. **(B)** The number of genes significantly DE when comparing M1, M1NR, and M2 to WB are shown as well as the number of those with fold changes over 2. **(C)** An Euler diagram visualizing the overlaps between the DE proteins for those three lines is presented with the upper number reporting upregulated genes and the lower number downregulated genes (different number colors were chosen to enhance contrast) and in panel **(D)** a selection of GO-term groups and the direction of their changes is shown for M1, M2, and the genes that are changed in M1 and reversed in M1NR.

As a next step in the characterization of MTZ resistant lines, we analyzed their transcriptomes by RNAseq during growth in the absence of MTZ (Figure 2). The transcriptomes of the two MTZ resistant lines and the revertant were compared to the transcriptome of the wild-type strain WB-C6. The two MTZ resistant lines clustered is distinct regions in PCA plots, while the two MTZ sensitive lines, WB-C6 and M1NR, showed considerable overlap (Supplementary Figure 5). We detected relatively small overall changes in the transcriptomes in regard to amplitudes of expression (largest gene expression differences in the high cysteine membrane proteins HCMPs and VSPs, as noted in earlier studies (Ansell et al., 2017; Müller et al., 2019)) but a large number of significantly differently expressed (DE) genes between the MTZ resistant isolates and the WB-C6 strain (Figures 2A,B). For M1NR we only found a total of 129 DE genes, in line with the revertant phenotype of this line (Figures 2B,C), with 29 genes downregulated more than twofold and 6 genes upregulated more than twofold (Supplementary Table 5). The numbers were substantially higher for the two resistant lines with 1454 DE genes for M1 and 1628 DE genes for M2, respectively (Figures 2B,C). Most changes were small and only 107 and 175 DE genes were upregulated or downregulated more than twofold in the M1 and M2 lines, respectively (Figure 2B and Supplementary Table 2). An Euler diagram of all DE genes showed that the two MTZ resistant lines have more DE genes in common than either of them have with the revertant. Around half of the genes that are DE are shared between the resistant lines, while the other half is unique for each line (Figure 2C and Supplementary Table 4). A set of 16 genes is upregulated in all three lines when comparing to the control levels of WB-C6, another 19 are downregulated in all (Supplementary Table 4).

To interrogate whether certain molecular functions were enriched, we analyzed the DE genes by GO-terms. For downregulated genes, no over-representation was observed for any of the lines (Figure 2D). For upregulated genes, M1 showed an enrichment in translation factor activity, whereas M2 displayed an enrichment of three molecular functions: kinases, oxidation-reduction processes, and cofactor binding. Scrutinizing genes that were DE in M1 but not in the M1NR revertant, a total of 721 were upregulated and 639 downregulated (Supplementary Table 2). GO-terms enriched in upregulated genes were connected to nucleic acid binding and metal cluster binding, whereas downregulated genes clustered in protein binding, transmembrane transport, and ligase activity. All GO-terms are summarized in Supplementary Table 6. Taken together, we observed heterogenous differences in transcriptomes associated to MTZ resistance while differential transcript levels were comparably small and that reversion of MTZ resistant tracked with a return to the levels in the MTZ sensitive parental cells.

### Metronidazole Resistance at the Protein Level Is Characterized by Changes in Oxidation-Reduction Processes and Stress Response Systems

**FIGURE 3:**
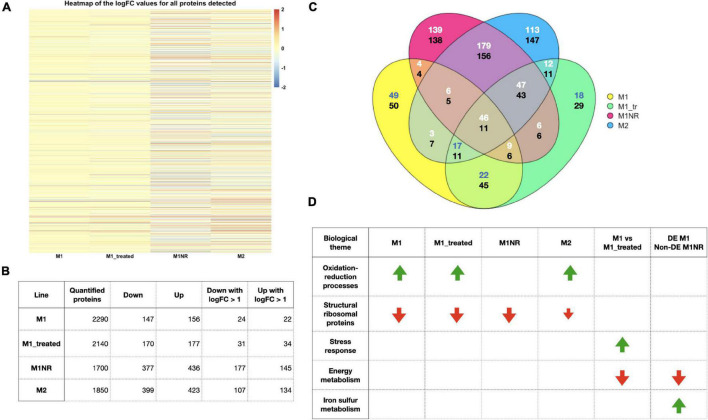
Overview of the data from our proteomics analysis. **(A)** A heatmap of the log2 fold changes for all proteins detected based on the comparisons, M1, M1_treated, M1NR, and M2 with WB is shown. A cut-off of 2 was used to enhance visualization of smaller fold changes. **(B)** The number of proteins quantified, significantly DE when comparing M1, M1_treated, M1NR, and M2 to WB and those with fold changes over 2 are shown. **(C)** A Venn diagram visualizing the overlaps between the DE proteins for those four datasets is presented with the upper number reporting upregulated genes and the lower number downregulated genes (different number colors were chosen to enhance contrast) and panel **(D)** a selection of GO-term groups and the direction of their changes is shown for M1, M1_treated, M1NR, and M2 and the genes that are changed in M1 and reversed in M1NR as well as those changed in M1_treated but not in M1.

To determine if the identified changes in the transcriptomes were reflected in the proteomes of the parasites, we analyzed the same lines using proteomics. As an additional level of information, we examined M1 both with and without MTZ added to the media to determine whether any responses were triggered by the mere presence of MTZ. In general, sample replicates clustered closer to each other than to other samples in PCA plots based on normalized LFQ values. This was however not the case for M1 and M1+MTZ, which clustered very close to each other in PCA plots and distance heatmaps (Supplementary Figure 6). Between 1767 and 2454 proteins could be detected with at least one peptide in three or more replicates for the different lines and were included in the analysis. Of these, 1700 could be quantified in comparisons between M1NR and WB-C6, 2290 for M1 and WB-C6, 2140 for M1+MTZ and WB-C6 and 1850 for M2 and WB-C6. 1589 proteins, roughly one third of the of the total parasite proteome, were detected and could be quantified in all lines. As can be seen in the heatmap of logFC values most proteins were found to not be DE when compared to WB-C6 (Figure 3A). For M1NR a total of 813 proteins were significantly DE (*p*-value = 0.05), for M1 this number is 303, for M1+MTZ 347 and for M2 822, respectively (Figure 3B and Supplementary Table 3). This observation was surprising since RNA levels revealed a different picture, with most genes DE in M1 and M2, but less in M1NR. 179 upregulated and 156 downregulated DE proteins were common between M1NR and M2, while the overlaps between M1NR and M1, as well as M2 and M1, were smaller (Figure 3C and Supplementary Table 7). In the comparison between M1 and M1+MTZ it is worth to note that while the overall number of DE proteins when compared to WB-C6 was comparable, only about 50% of proteins were represented in both. Lastly, a total of 17 proteins were upregulated and 11 downregulated more than twofold in the resistant lines and not in the revertant M1NR (Table 2).

**TABLE 1:** mRNAs regulated in a similar manner in the resistant isolates M1 (treated and untreated) and M2 but not in the revertant M1NR.

Protein (Gene id)	Level of DE compared to WB-C6 (log2)			
	M1NR	M1	M1_tr	M2
GL50803_16076-Peroxiredoxin 1ai	0	1.14	1.01	1.74
GL50803_17516-Cathepsin B	0	0.84	0.44	0.78
GL50803_17476-High cysteine membrane protein	0	0.84	1.06	0.59
GL50803_103992-VSP	−0.81	0.67	1.32	3.6
GL50803_103713-Protein disulfide isomerase PDI4	0	0.63	0.61	0.51
GL50803_103944-Kinase, NEK	0	0.62	0.71	1.69
GL50803_14519-Iron-sulfur cluster biosynthesis protein IscS	0	0.61	0.59	0.9
GL50803_8329-Vacuolar protein sorting 25	0	0.54	0.47	0.31
GL50803_114852-High cysteine membrane protein VSP-like	0	0.52	0.89	1.32
GL50803_16471-Ankyrin repeat protein 1	0	0.51	0.51	0.6
GL50803_24372-ENC6 protein	0	0.4	0.46	0.45
GL50803_6242-Translationally controlled tumor protein-like protein	0	0.36	0.39	0.84
GL50803_11129-Hypothetical protein	0	0.35	0.35	0.36
GL50803_5800-Lipid binding protein	0	0.33	0.76	0.71
GL50803_17046-Ankyrin repeat protein 1	0	–0.44	–0.68	–0.6
GL50803_95549-Kinase, NEK	0	–0.52	–0.93	–1
GL50803_5845-Ribosomal protein S8	0	–0.54	–0.75	–0.58
GL50803_11599-Acid phosphatase	0	–0.62	–0.76	–0.57
GL50803_16265-Ribosomal protein S10a	0	–0.63	–0.72	–0.98
GL50803_11299-Amino acid transporter, putative	0	–1.05	–1.07	–1.15
GL50803_10524-Hypothetical protein	0	–2.08	–1.99	–1.45
GL50803_14586-VSP with INR	1.87	–5.4	–5.38	–2.29

**TABLE 2:** Proteins regulated in a similar manner on the protein level in resistant isolates M1 (treated and untreated) and M2 but not in the revertant M1NR.

Protein (Geneid)	Level of DE compared to WB-C6 (log2)					
	M1NRr*	M1NRp	M1r	M1p	M2r	M2p
GL50803_103992-VSP	–2.92	–0.81	1.03	0.67	1.39	3.60
GL50803_112113-VSP	0.00	NA	2.02	1.95	3.38	4.18
GL50803_6242-Translationally controlled tumor protein-like protein	0.00	0.00	0.71	0.36	0.50	0.84
GL50803_4410-SALP-1	0.00	0.00	–0.29	–0.67	–0.61	–0.65
GL50803_16076-Peroxiredoxin 1ai	0.00	0.00	1.05	1.14	1.54	1.74
GL50803_14456-Low molecular weight protein-tyrosine-phosphatase	0.00	NA	0.58	0.32	0.85	0.63
GL50803_14519-Iron-sulfur cluster biosynthesis protein IscS	0.00	0.00	0.68	0.61	0.84	0.90
GL50803_10524-Hypothetical protein	0.00	0.00	–0.49	–2.08	–0.74	–1.45
GL50803_7715-High cysteine membrane protein Group 1	0.00	NA	0.28	0.85	0.62	1.06

*r, RNAseq data; p, proteomics data.

As post-translational modifications (PTMs) were previously linked to MTZ resistance in *Giardia* (Emery et al., 2018), we also analyzed the samples for these. For PTMs reported in the past to be changed (i.e., lysine acetylation, lysine mono-methylation, and phosphorylation), none could be linked to MTZ resistance in the lines analyzed herein (Supplementary Table 3).

Further, we analyzed for potential GO-term enrichments among DE proteins to find common themes (Figure 3D and Supplementary Table 6). Here some interesting patterns emerged: In all three resistant datasets, oxidation-reduction processes and processes connected to oxidative stress were upregulated, while this was not the case for M1NR. One group of proteins that was downregulated for all lines were ribosomal proteins. When analyzing the results for M1 vs. M1+MTZ, we found stress response proteins upregulated in presence of MTZ, while among the proteins downregulated GO-terms in connection to ribosomal proteins, glucose metabolism, and nucleic acid binding were enriched. When looking at the comparison with M1NR and M1 without MTZ, we found GO-terms connected to iron-sulfur metabolism and metallopeptidases to be upregulated and those connected to the ribosome and RNA binding were downregulated.

### Correlations Between Transcript and Protein Abundances From Genes With Possible Roles in Metronidazole Resistance

**FIGURE 4:**
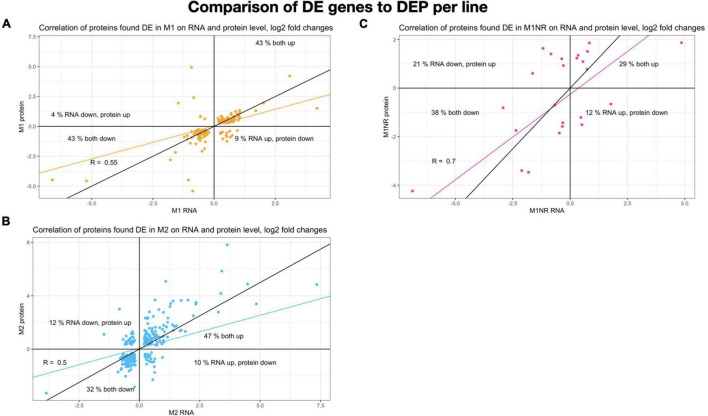
Comparison of genes and proteins changed for lines M1, M1NR, and M2. Log2 fold changes for all genes present in both datasets are plotted with the changes on RNA level on the *X*-axis and the protein changes on the *Y*-axis. A correlation is calculated and presented in the same color, the black line presents a correlation of one. Percentages are given for each quadrant to show how many of the proteins fall into the same quadrant. **(A)** Data for M1 is shown, **(B)** for M1NR, and **(C)** for M2.

To gain further information about gene expression changes in the MTZ resistant lines studied here, we compared if DE genes were regulated in a similar manner on transcript and protein levels. Included in this analysis were genes that showed DE in both the transcriptomic and proteomic data sets, independent of directionality. This resulted in 24, 134, and 247 genes for M1NR, M1, and M2, respectively. To examine whether these correlated in directionality, the logFC values were plotted against each other (Figure 4). For the M1NR line roughly two thirds of the 24 proteins found to be DE in proteomics and transcriptomal analyses were regulated in the same way as the corresponding genes, while those values were higher for the resistant lines at 86% for M1 and 79% for M2. Thus, we find that in our expression analyses changes on RNA and protein level are generally well correlated. Among the genes found to be DE in the same direction in the two MTZ resistant lines, but not in M1NR (Supplementary Table 7), we noticed a total of nine, among which there was one high cysteine membrane protein, two VSPs, one hypothetical protein, one iron-sulfur cluster protein, one tyrosine phosphatase, peroxiredoxin 1ai, the adhesive disc protein SALP-1 and one tumor-like protein.

### Meta-Analysis Reveals Different and Common Themes in Metronidazole Resistance Associated Gene Expression

**FIGURE 5:**
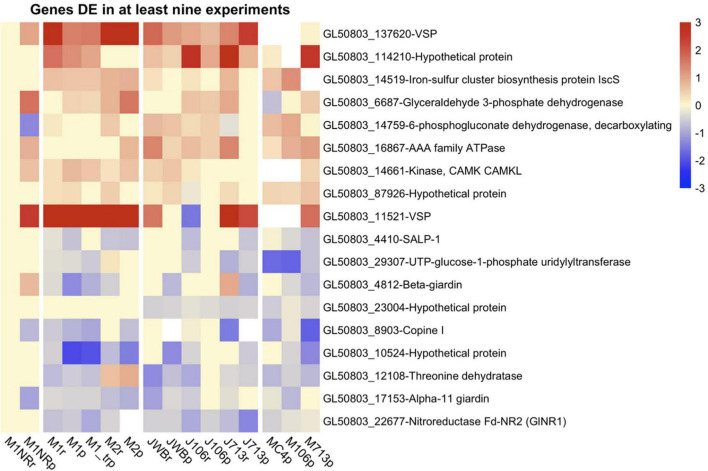
Heatmap of log2 fold changes for genes changed in at least nine datasets from resistant lines. Line names ending in r mark RNA datasets, lines names ending in p proteomics datasets. Datasets produced in the Jex lab are named with a starting J, while those from the Müller lab have a starting M. Fold changes were capped at 3 to enable comparison between different heatmaps and visualization of small changes. For every gene presented both the Geneid (starting with GL50803) and the annotated function are given. Red means a gene is upregulated when compared to the respective wildtype, while blue shows a down-regulation.

As the next step, we included additional datasets on MTZ resistant *G. intestinalis* lines created and analyzed in other laboratories for correlative purposes and to broaden the view of gene regulatory associations with MTZ resistance. First we used the complete transcriptomics dataset on three MTZ resistant lines from Ansell et al. (2017). In order to make the data sets comparable, we first had to re-analyze their data set, before comparing DE genes across all five MTZ resistant lines. These comparisons resulted in 39 upregulated and 21 downregulated genes shared between all MTZ resistant lines (Supplementary Tables 8, 10). Among the upregulated genes were twelve VSPs and two HCMPs, ten hypothetical proteins and six kinases (Supplementary Table 10). Among the downregulated genes were seven hypothetical proteins, four ankyrin repeat proteins, three alpha-giardins and two oxidoreductases (GL50803_17151 and 22677, Supplementary Table 10). None of these the genes had changed expression in the revertant M1NR. We also performed a similar meta-analysis on proteomic findings from the same parasite lines (Emery et al., 2018) as well as lines analyzed by Müller et al. (2019; Supplementary Tables 9, 10). No DE proteins were shared in between all parasite lines but two were upregulated in six out of seven lines: one VSP (GL50803_137620) and a hypothetical surface protein, GL50803_114210 (Supplementary Table 10). Eight proteins showed down-regulation in most of the MTZ resistant isolates (Supplementary Table 10). GlNR1 (Gl50803_22677) was downregulated in all lines but in M2, (Figure 5 and Supplementary Table 10), where it could only be detected at a low level in one of the four proteomic replicates resulting in removal from the DE analyses due to our inclusion criteria of detection in at least two replicates; this shows that GlNR1 has even lower expression in M2 than in any other line. Other proteins are GL50803_11359, ribosomal protein S4 and alpha-11 giardin, which are downregulated in all isolates, including 106r and 713r in the Müller dataset but not in the same isolates in the Emery dataset. We also found one hypothetical protein (GL50803_10524), localized to the adhesive disc, downregulated in all lines but 106r. Another four proteins are downregulated in five or four out of the six lines: Ribosomal protein S8 (GL50803_5845), a threonine dehydratase (GL50803_1210), an alanyl dipeptidyl peptidase (GL50803_15574) and the adhesive disc protein beta-giardin (GL40803_4812). From the genes with differential expression detected on both transcript and protein levels, we identified nine to be generally upregulated and nine to be mostly downregulated (Figure 5). One gene clearly stood out among these, namely GlNR1, which was detected as downregulated in all resistant lines, except on protein level in M2 as explained above.

### Supervised Examination of Gene Groups Potentially Involved in Conferring Metronidazole Resistance

Since few genes that were identified by transcriptomics and/or proteomics are known to be involved in MTZ resistance in all six MTZ resistant *G. intestinalis* lines examined to date, we decided to have a closer look at the groups of genes implied in the literature or by our GO-term analysis. Among those named in the literature were genes involved in attachment (Tejman-Yarden et al., 2011), energy metabolism (Müller et al., 2007, 2019; Tejman-Yarden et al., 2011; Begaydarova et al., 2015), as well as genes involved in encystation (Einarsson et al., 2016b). Another group of interesting genes are those involved in oxidoreduction processes. For all gene groups, we defined a list and report expression level changes in all resistant lines on both transcriptomic and proteomic level. The complete lists with all expression data can be found in Supplementary Table 11.

**FIGURE 6:**
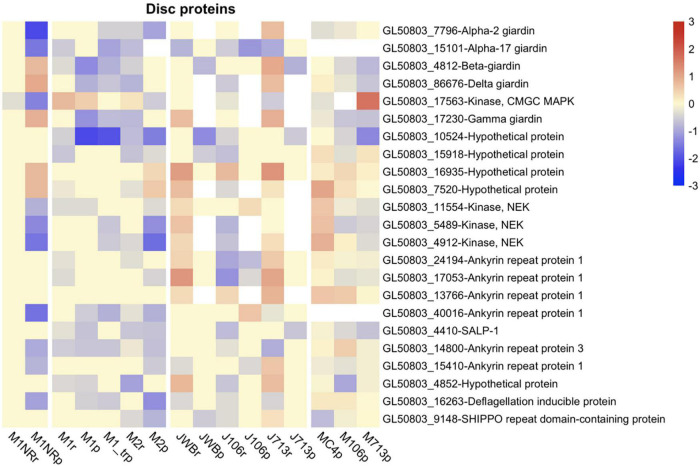
Heatmap of log2 fold changes for attachment related genes changed in at least six datasets from resistant lines. Line names ending in r mark RNA datasets, lines names ending in p proteomics datasets. Datasets produced in the Jex lab are named with a starting J, while those from the Müller lab have a starting M. Fold changes were capped at 3 to enable comparison between different heatmaps and visualization of small changes. For every gene presented both the Geneid (starting with GL50803) and the annotated function are given. Red means a gene is upregulated when compared to the respective wildtype, while blue shows a down-regulation.

The first group that we examined were adhesive disc-associated proteins (DAPs) since attachment is affected in the M1 and M2 lines (Tejman-Yarden et al., 2011). We based our selection of 87 DAPs studied by Nosala et al. (2020). We found that DE disc proteins are more often downregulated in M1 and/or M2 in comparison to the other resistant lines, with two, SALP-1 and GL50803_10524, being downregulated in both lines but not changed in the revertant M1NR (Figure 6). In the other three resistant lines no clear trend for DE of the DAPs could be seen, even though many of the proteins were DE (Figure 6 and Supplementary Table 11). This includes SALP-1 and GL50803_10524 and the well-characterized disc protein beta-giardin (Pathuri et al., 2007; Kim et al., 2013) (Figure 6). Thus, some of the main disc proteins are downregulated in several MTZ resistant lines.

We next analyzed glucose metabolism, since this pathway was reported to be disturbed in the mutants M1 and M2 (Tejman-Yarden et al., 2011). No clear picture emerged but two proteins showed up-regulation in several of the resistant lines: Fructose-bisphosphate aldolase (GL50803_11043) and one of the two giardial glyceraldehyde 3-phosphate dehydrogenases (GL50803_17043) (Supplementary Table 11). In the fermentation pathways following on glycolysis, no single gene is regulated in a similar manner in all resistant lines (Supplementary Table 11). The same is true for the arginine dehydrolase pathway (Supplementary Table 11). The PFORs themselves are downregulated in a few of the resistant lines but unchanged in others: PFOR 1 is downregulated in WBr (RNA and protein), 106r (RNA) and in M2 (protein). PFOR 2 is downregulated in M2 and WBr. Interestingly one gene that is catalyzing production of one substrate to PFOR 2 – threonine dehydratase (GL50803_12108) – is downregulated in most resistant lines (Supplementary Table 11). Overall, gene expression changes can be seen in energy generating pathways in the resistant mutants but none of the changes are consistently observed in all MTZ resistant lines.

**FIGURE 7:**
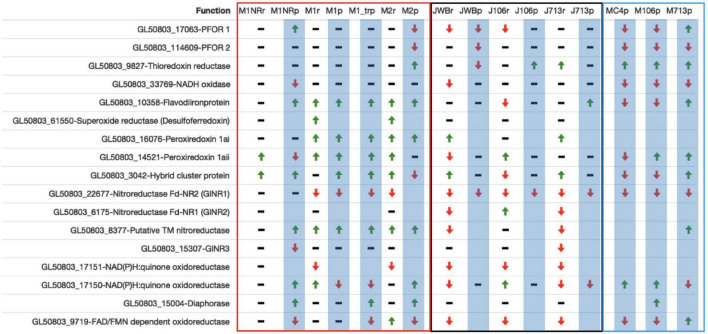
Datatable of a selection of oxidoreductases. Line names ending in r mark RNA datasets, lines names ending in p proteomics datasets and have a blue background. Datasets produced in the Jex lab are named with a starting J, while those from the Müller lab have a starting M. Datasets produced by us are within the red frame, Jex datasets are framed black while datasets from the Müller lab are framed blue. For every gene presented both the Geneid (starting in GL50803) and the annotated function are given. A green arrow upward means a gene is upregulated when compared to the respective wildtype, a black bar means no changes, a red arrow downward shows down-regulation and an empty field means that the protein was not detected in this dataset.

Finally we examined genes involved in oxidoreductive processes as they are involved in MTZ activation (Dingsdag and Hunter, 2018) and since a lowered level of oxygen consumption has been observed in MTZ resistant *Giardia* lines. With our new data and genes previously implicated in resistance (Ansell et al., 2017; Saghaug et al., 2019) we compiled a gene candidate list in Supplementary Table 11. A summary of data from selected genes can be found in Figure 7. Neither PFOR 1 (GL50803_17063) nor PFOR 2 (GL50803_114609) are consistently DE in the resistant lines, but tend to be downregulated (Figure 7). Thioredoxin reductase is upregulated in several lines (GL50803_9827, Figure 7). NADH oxidase (GL50803_33769) is mostly downregulated on protein level. The flavodiironprotein GL50803_10358 is upregulated in some lines and downregulated in others. Superoxide reductase (GL50803_61550) could not be found on protein level in any line. On RNA level it is upregulated in M1 and M2 (Figure 7). For the three intracellular proteins with peroxiredoxin function (GL50803_16076, GL50803_14521 and GL50803_3042) the general trend shows an up-regulation of these proteins, especially in the lines examined in this study (Figure 7). The next group are the nitroreductases (Figure 7). Here GlNR1 (GL50803_22677) shows the clearest profile with down-regulation in all lines but M2, where it could not be detected on protein level (Figure 7). GlNR2 (GL50803_6175) could not be found on protein level and behaves inconclusively on RNA level (Figure 7). The putative transmembrane NR (GL50803_8377) is upregulated in all experiments for our lines and in 713-r in the Müller study, downregulated on RNA level for WBr and 713-r and not changed in 106-r while not being found in the remaining protein samples (Figure 7). GlNR3 (GL50803_15307), finally, is downregulated on RNA level in 713-r and on protein level in M1NR (Figure 7). The group of quinone reductases show a mixed profile: Gl50803_17151 is downregulated on RNA level for all resistant lines and not found on protein level for either of them (Figure 7), whereas regulation of GL50803_17150 is inconclusive (Figure 7). The DT diaphorase (GL50803_15004) is only DE – upregulated – in M1NR and M1_treated, M2 and 106-r (Müller study) on protein level and otherwise unchanged (Figure 7). The FAD/FMN dependent oxidoreductase GL50803_9719 is more often downregulated than upregulated. Taken together, the different resistant isolates have unique oxidoreductase expression profiles with two notable exceptions: GlNR1 and the quinone reductase Gl50803_17151 are consistently downregulated.

## Discussion

Metronidazole resistance in *G. intestinalis* and other pathogens is increasing (Carter et al., 2018). A comprehensive view of the mechanisms that drive and counteract resistance development is needed. The drug resistance appears in many cases to depend on non-genetic changes (Saghaug et al., 2019), an easily reversible adaptation that can potentially develop rapidly compared to fixation of mutations and other changes to the genome. This presents obstacles for proper surveillance of the spread of resistant populations, which in turn limits the possibility to manage infections. In the case of *G. intestinalis*, evolution of MTZ resistance appears to be strongly associated with a significant and multimodal fitness cost (reduced growth rate, attachment, encystation, and animal infectivity), which might explain why it took decades for drug resistance to develop. Although reduced encystation efficiency was noted in the M1 and M2 isolates studied here, the underlying molecular rationale is not known as it is still unclear how encystation is initiated at the molecular level (Rojas-López et al., 2021). The encystation process is complex, as evidenced by the extensive and coordinated gene regulatory program that is associated to formation of viable cysts, which holds true for the early part of the process as well (Rojas-López et al., 2021). Thus, the substantial transcriptomic and proteomic changes the parasite needs in order to tolerate MTZ may be in their sum too costly to afford creation of transmission stages. The reduced attachment of M1 and M2 is directly correlated to down-regulation of several adhesive disc proteins (Figure 6). Interestingly, all the fitness cost related phenotypes could be reversed by growth in absence of drug, as shown by the revertant M1NR, which was also paralleled by a resetting of gene expression. It is important to note however, that the M1 and M2 mutants were generated using UV-induced mutagenesis and selection in MTZ, which was not the case with the other studied isolates (Ansell et al., 2017; Müller et al., 2019). This might explain why there are certain phenotypes that are stronger in these two isolates.

**FIGURE 8:**
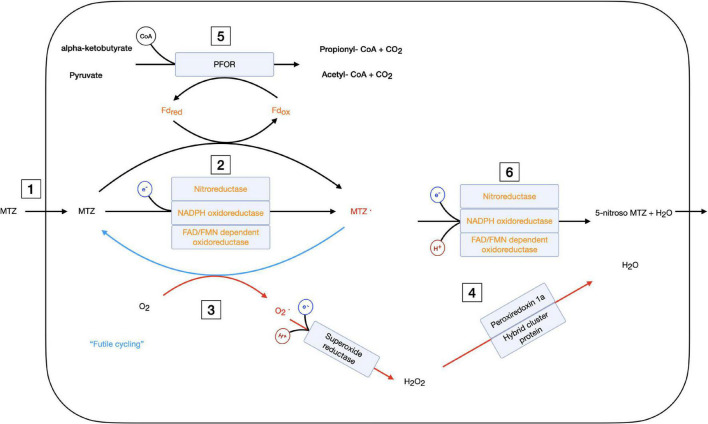
Schematic overview of cellular mechanism involving metronidazole, oxygen, and quinones in the giardial cell. Metronidazole activating enzymes and molecules are shown in orange text, radicals are shown in red. Red arrows show reduction of oxygen, superoxide and hydrogen peroxide and blue arrows denote futile cycling. Electrons entering the reactions are shown in a blue circle, hydrogen protons in red and CoA (Coenzyme A) in black. Several enzymes on the same reaction arrow show that either of these enzymes can catalyze this reaction. The cellular metabolism of metronidazole is given with possible resistance mechanisms marked by numbers: **1** – reduced uptake, **2** – reduced activation by down-regulation of activating enzymes, **3** – increased futile cycling by increased oxygen content, **4** – upregulated oxygen stress response, **5** – reduced electron flow through PFOR resulting in reduced activation by Fd, and **6** – fastened deactivation of metronidazole radical. MTZ, metronidazole; Fd, ferredoxin; red, reduced; ox, oxidized; PFOR, pyruvate ferredoxin oxidoreductase; CoA, coenzyme A.

The data generated here, together with earlier data, suggest that MTZ resistance in *Giardia* is mechanistically multifactorial (Figure 8). Several different MTZ resistance mechanisms have been suggested, including reduced uptake, increased efflux, decreased activation, increased cellular levels of oxygen, increased oxygen defenses, and DNA repair (Figure 8). MTZ resistant *Giardia* isolates selected *in vitro* display different patterns of DE genes with important roles in the different resistance mechanisms (Figure 8). This suggest that different isolates combine different mechanisms and the sum is generating the resistance phenotype and associated phenotypes. In the following paragraphs, we have put the most significant DE genes in context with several different putative resistance mechanisms.

### Alterations in Outer Membrane Structure

Metronidazole is believed to be mostly taken up via passive diffusion (Dingsdag and Hunter, 2018) and defects in MTZ transport, accompanied by changes in cell wall structure, have been described in other systems, such as *B. fragilis* (Britz and Wilkinson, 1979). It is plausible that changes in the outer membrane of *Giardia* similarly could affect MTZ uptake. In line with this, one hypothetical membrane protein (GL50803_114210) and members from three multigene families, encoding proteins with surface membrane association, repeatedly show differential gene expression in MTZ resistant lines: The variable surface proteins (VSPs) (Gargantini et al., 2016), High-cysteine membrane proteins (HCMPs, (Peirasmaki et al., 2020)) and the alpha-giardins (Weiland et al., 2005). The VSPs have also been suggested to change the membrane dynamics of *Giardia* trophozoites (Svärd et al., 1998; Singer et al., 2001; Sterk et al., 2007; Mendez et al., 2015) and specific HCMPs are upregulated upon host cell interactions, oxidative stress, and encystation (Einarsson et al., 2015; Ma’ayeh et al., 2015; Peirasmaki et al., 2020). Both families were implied to play a role in MTZ resistance in *G. intestinalis* in earlier characterizations of MTZ resistant lines (Müller et al., 2007). The problem with these genes is that *Giardia* trophozoites naturally change VSP and HCMP expression rapidly due to their role in antigenic variation. Nonetheless, two VSPs (GL50803_11521 and GL50803_137620) are consistently upregulated in most MTZ resistant lines (Figure 5). Further studies of these two specific VSPs and GL50803_114210 and their effect on MTZ sensitivity will be interesting.

The Ca^2+^ and phospholipid-binding alpha-giardins are involved in many cellular functions involving the cell membrane, such as permeability, exo- and endocytosis, membrane scaffolding, and trafficking (Weiland et al., 2005). One particular alpha-giardin, Alpha-11 giardin (GL50803_17153) (Pathuri et al., 2007), has been shown to localize to plasma membranes and basal bodies of anterior flagella in trophozoites (Kim et al., 2013) and has also been shown to bind to metronidazole and tinidazole (Leitsch et al., 2012). Interestingly, it is highly expressed in wildtype trophozoites, whereas it is downregulated in most resistant lines. Thus, there are several lines of evidence that support associations between changes to the membrane associated proteome and MTZ resistance, changes that could be involved in reduced uptake, increased efflux or protection against MTZ induced oxidative stress (Ansell et al., 2017).

### Reduced Drug Activation

Metronidazole is a pro-drug that must be reduced to reactive cytotoxic derivatives (Dingsdag and Hunter, 2018). In many different organisms, oxidoreductases are the main activators of nitroimidazole drugs, even though they can be activated by reductive agents as well (Chen and Kay Blanchard, 1979). The most commonly described MTZ resistance mechanism in bacteria is decreased activity of oxidoreductases via mutations (Dingsdag and Hunter, 2018). Therefore, investigation of MTZ resistance in *G. intestinalis* has also focused on oxidoreductases as well as other oxidative and reductive enzymes and several candidates have been brought forward over the years (Townson et al., 1996; Leitsch et al., 2011; Nillius et al., 2011). Apparent from our analysis of M1, M2 and the revertant M1NR, oxidation and reduction are among the most prominent biological processes that are altered in association with MTZ resistance in these isolates. Scrutinization of individual enzymes and comparisons with the other resistant strains reveal a heterogenous picture, with changes in certain putative activating enzymes being shared by many whereas others are strain-specific (Figure 7).

Nitroreductases have been suggested to be the major activating enzymes of metronidazole in *Giardia* (Nillius et al., 2011; Müller et al., 2013, 2015; Müller and Müller, 2019; Thomas and Gwenin, 2021). The oxygen-insensitive *Giardia* nitroreductase-1 (GlNR1, GL50803_22677) has an FMN-binding domain and a ferredoxin-like domain with a predicted FeS cluster and it is the most consistently downregulated gene in MTZ resistant lines (Figure 7). It can render over-expressing *Giardia* and *E. coli* more sensitive to MTZ (Nillius et al., 2011). However, a study of recombinant GlNR1 failed to show reduction of MTZ *in vitro* (Müller and Müller, 2019), which might be due to not fully active enzyme from *E. coli* or the lack of cellular co-factors needed for activity. The cellular function of GlNR1 is not known but it has greater affinity for quinones over nitroimidazoles, suggesting a role in quinone reduction. It co-purifies with several other enzymes mostly from energy pathways as well as with nitroreductase GlNR2 (Müller et al., 2015). Furthermore, GlNR1 has been reported to be downregulated in nitroimidazole resistant lines by most publications to date (Ansell et al., 2017; Emery et al., 2018).

The second nitroreductase, GlNR2 (GL50803_6175), has been suggested to have detoxifying activity (Müller and Müller, 2019). Structurally it is similar to GlNR1 but overexpression of NR2 in *Giardia* increases the tolerance to MTZ and overexpression in *E. coli* results in total insensitivity to the drug (Müller et al., 2013; Müller and Müller, 2019). It does not only co-purify with GlNR1 but the two proteins interact in a synergistic way for substrate reduction. Increased expression of GlNR2 could be an active resistance mechanism but overexpression of GlNR2 is not seen in the seven MTZ resistant isolates generated *in vitro* (Figure 7).

A third nitroreductase, GlNR3 (GL50803_15307), lacks the ferredoxin domain and can reduce quinones in a similar manner to both other nitroreductases albeit very slow (Müller and Müller, 2019). Overexpression in *E. coli* shows a significantly increased sensitivity to MTZ, like GlNR1, but over-expression in *Giardia* has no effect (Müller and Müller, 2019) and no changes are seen in the resistant isolates here (Figure 7). The final nitroreductase (GL50803_8377) is more distantly related to the other three nitroreductases. It is uncharacterized to date but it has a predicted FMN-dependent nitroreductase domain and it is upregulated in the mutants M1 and M2 and 713 in the Müller dataset (Figure 7).

The two quinone reductases (GL50803_17150 and GL50803_17151) were until recently less characterized. Müller et al. (2021) showed that GL50803_17150 can reduce quinones and nitro compounds and prefer NADPH over NADP. They also found that is closely related to the diaphorase in structure with an 86% sequence identity. Some isolates show up-regulation whereas others show down-regulation of GL50803_17150 but GL50803_17151 is downregulated in all studied MTZ resistant isolates (Figure 7). Not much is known about GL50803_17151, except that it is highly transcribed but poorly translated and has about 40% sequence identity to diaphorase.

Proteins with PFOR activity were early candidates for MTZ activation in *Giardia* (Townson et al., 1996). PFORs are involved in amino acid, fatty acid and glycolytic metabolism in *Giardia*, and transfer electrons to ferredoxins, small iron-sulfur proteins involved in electron transfers in many metabolic reactions (Ginger et al., 2010). The *G. intestinalis* WB genome encodes two PFOR paralogs, PFOR 1 and 2, and PFOR2 is downregulated on the protein level in most MTZ resistant isolates (Figure 7). We also found indirect evidence for a change of electron flow through PFOR 2 via down-regulation of threonine dehydratase (GL50803_12108). Another putative activating oxidoreductase is thioredoxin reductase (GL50803_9827). This enzyme is important in redox-regulation in *Giardia* and has been shown to reduce and thereby activate MTZ with flavin adenine dinucleotide (FAD) as a cofactor (Leitsch et al., 2011, 2016). In our expression analyses, we observed an up-regulation on the protein level of thioredoxin reductase in most MTZ resistant isolates but earlier data suggested reduced reduction of FAD and reduced FAD pool sizes in several MTZ resistant lines (Leitsch et al., 2011; Müller et al., 2018), so a better direct indicator would be to measure thioredoxin reductase activity in the isolates.

To summarize, the level of down-regulation of putative MTZ activating oxidoreductases in *Giardia* on the protein level is not high but the combination of a down-regulation of several activating enzymes in specific isolates, together with reduce levels of crucial co-factors, could reduce the activation levels of MTZ enough to obtain tolerance to the drug (Figure 8).

### Increased Cellular Oxygen Levels and Up-Regulation of Oxygen Stress Responses

High cellular levels of oxygen protect organisms against MTZ. It has been proposed that reoxidation of the nitroradical anion regenerates the MTZ prodrug in a process called futile cycling (Figure 8) and that this is the main reason for the selectivity of MTZ for anaerobic cells (Dingsdag and Hunter, 2018). Increased oxygen levels can also be a resistance mechanism (the aerobic resistance type) and resistant isolates of the pathogenic protozoan *Trichomonas vaginalis* have higher intracellular oxygen concentrations than the wild-type cells, leading to deactivation of the drug and tolerance (Leitsch et al., 2014a). However, the presence of oxygen generates oxygen radicals that may induce DNA damage which the resistant cells need to deal with.

Two proteins involved in oxidative stress regulation in *Giardia* are NADH oxidase (GL50803_33769) and the flavodiironprotein (FDP) GL50803_10358 (Jiménez-González et al., 2019). The NADH oxidase has been studied intensively and can catalyze the direct conversion of O_2_ to water without creating a superoxide radical. While it can use other electron acceptors, it cannot reduce MTZ (Brown et al., 1996). In accordance with this, we see almost no DE in the MTZ resistant *Giardia* lines. Interestingly, FDP has been reported to catalyze the same reaction as the NADH oxidase and it is upregulated in most examined MTZ resistant lines (Figure 7). The main difference in the reactions is that FDP uses rubredoxin as cofactor instead of NAD(P)H. No rubredoxin has been found in the giardial genome so far (Di Matteo et al., 2008) so Jiménez-González et al. (2019) have proposed that one of the six ferredoxins might provide the electrons, thereby connecting this enzyme to the PFOR pathway. Up-regulation of FDP might hence work as a non-MTZ electron sink for the cell.

Superoxide radicals are rapidly converted to hydrogen peroxide by a superoxide reductase (SOR-GL50803_61550) of bacterial origin in *G. intestinalis* (Testa et al., 2011; Mastronicola et al., 2016). The source of the reducing equivalents for SOR is currently not known. SOR also often uses rubredoxin as electron donor so one of the six ferredoxins might be filling this function as well. Peptides from SOR are not found in any of the proteomics datasets (Figure 7), but it is it is upregulated on RNA level in M1 and M2. In case of an “aerobic resistance type” up-regulation of this enzyme would help detoxify additional superoxide radicals. The hydrogen peroxide produced by this enzyme and others is detoxified by four proteins with peroxiredoxin activity in *G. intestinalis* of which three are located in the cytoplasm (Schneider et al., 2011; Mastronicola et al., 2014; Ma’ayeh et al., 2017): Peroxiredoxin 1ai (GL50803_16076), peroxiredoxin 1aii (GL50803_14521) and HCP (GL50803_3042). They all catalyze the reaction H_2_O_2_ to H_2_O with the peroxiredoxins also being able to metabolize other alkylperoxides and peroxynitrite, molecules which are produced by superoxide radicals (Mastronicola et al., 2014). All three enzymes show up-regulation in most of the resistant lines (Figure 7). Up-regulation of these enzymes could help in attenuating the consequences of oxidative stress.

An “aerobic resistance type” with futile cycling finally seems possible, as we see a consistent up-regulation of enzymes that are involved in oxidative stress. Two studies looked at oxygen consumption in *Giardia*, with one finding no difference in the ability to deplete oxygen, but uptake speed of MTZ resistant lines being enhanced (Ellis et al., 1993) and the second finding that oxygen consumption was significantly reduced in a nitroimidazole resistant line (Müller et al., 2018). A next step could be to measure intracellular oxygen levels to see whether oxygen is enriched intracellularly in resistant lines.

### Increased DNA Repair

Several studies have shown that MTZ induces DNA damage in *Giardia* (Bagchi et al., 2012; Uzlikova and Nohynkova, 2014; Ordoñez-Quiroz et al., 2018) and the treated cells arrest in the S phase of DNA replication (Uzlikova and Nohynkova, 2014). It has been shown that single and double stranded DNA breaks and proteins in the DNA homologous recombination repair pathway (MC1B and Mre11) are transiently induced in *Giardia* after MTZ treatment (Ordoñez-Quiroz et al., 2018). Thus, an induction of DNA repair systems could reduce the damaging effects on DNA by MTZ-derived radicals (Figure 8). We studied the expression of DNA repair related proteins in *Giardia* in the resistant isolates on both the RNA and protein levels but could not see any changes (Supplementary Table 11).

## Conclusion

Metronidazole has long been a mainstay in the treatment of infections with anaerobic and microaerophilic pathogens. For most of the six decades since the development of MTZ, resistance was a small problem compared to other antibiotics, but in recent years this has changed. In this work, we have analyzed two MTZ resistant *G. intestinalis* lines for their phenotypic, transcriptomic, and proteomic changes, as well as how those changes are reversed in a revertant. We then compared our results to phenotypic, transcriptomic and proteomic findings published on four additional MTZ resistant lines, which were generated with different approaches in different laboratories from different parental strains. Based on the combined data from our study and prior work, we suggest the following model for MTZ resistance in *G. intestinalis*: MTZ enters the cell in its prodrug form and gets activated by one of the many oxidoreductases (Figure 8). There is clear evidence for a reduction of MTZ activation on different levels both in our dataset and reported in earlier studies as a cause of increased resistance to MTZ in *G. intestinalis*. Energy metabolism, pyruvate formation and fermentation and the pool of redoxmolecules like FAD in the cell also affect the activation of MTZ (Figure 8). The created radical is highly reactive, in contrast to semi-quinones and other natural substrates of the oxidoreductases (Supplementary Figure 7). MTZ radicals cause damage in a non-targeted manner (Uzlikova and Nohynkova, 2014; Dingsdag and Hunter, 2018; Lauwaet et al., 2020), especially to the DNA (Figure 8). In the presence of oxygen, radical formation is reduced by futile cycling (Figure 8). Induction of oxidative stress responses and DNA repair mechanisms reduce the effects of remaining MTZ radicals. There are putative oxidoreductases that can inactivate MTZ but it still needs to be proven if this is a true resistance mechanism in *Giardia*. Overall, this leads to a multifactorial presentation of MTZ resistance in *G. intestinalis*, with all changes together lowering the levels of active radicals enough for the pathogen to survive treatment.

## Data Availability Statement

The datasets presented in this study can be found in online repositories. The names of the repository/repositories and accession number(s) can be found below: https://www.ebi.ac.uk/pride/archive/, PXD027813 and https://www.ncbi.nlm.nih.gov/geo/, GSE139624.

## Author Contributions

SK performed the experiments, analyzed the data, and wrote the first draft of the manuscript. UR, YM, and LE analyzed part of the data and contributed to writing of the first draft of the manuscript. SS conceived and designed the experiments, analyzed part of the data, and wrote parts of the manuscript. All authors revised the final version of the manuscript.

## Conflict of Interest

The authors declare that the research was conducted in the absence of any commercial or financial relationships that could be construed as a potential conflict of interest.

## Publisher’s Note

All claims expressed in this article are solely those of the authors and do not necessarily represent those of their affiliated organizations, or those of the publisher, the editors and the reviewers. Any product that may be evaluated in this article, or claim that may be made by its manufacturer, is not guaranteed or endorsed by the publisher.

## Footnotes

1
www.scilifelab.se

2
https://www.ncbi.nlm.nih.gov/geo/query/acc.cgi?acc=GSE139624

3
https://giardiadb.org/giardiadb/app/downloads/release-50/GintestinalisAssemblageAWB/fasta/data/
