# Supplementary material for: Characterization of Metronidazole-Resistant Giardia intestinalis Lines by Comparative Transcriptomics and Proteomics
Source: Front Microbiol. 2022 Feb 10;13:834008. doi: 10.3389/fmicb.2022.834008 (PMC8866875; doi:10.3389/fmicb.2022.834008)
Supplement: Supplementary file 12 [file Data_Sheet_1.PDF]

Supplementary Figures

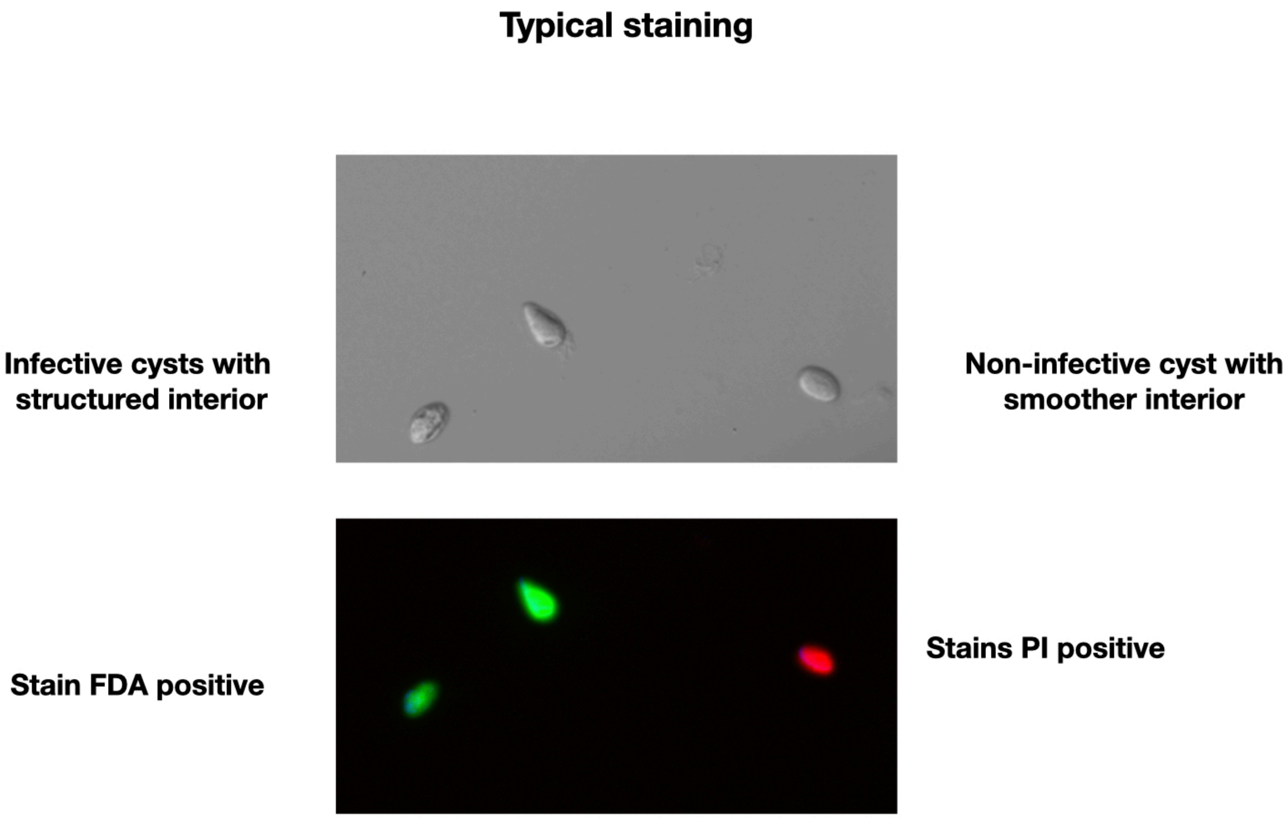

Supplementary Figure 1. Microscope images of typical staining of WB-C6 cysts. The cells have been encysted using the Uppsala encystation protocol and water treated for at least 72h before staining. Staining was done on ice for 20min in 100µL FDA working solution, 40µL of Propidium Iodide Ready Flow™ Reagent (Invitrogen) and 60µl PBS in a total volume of 200µl. Images were acquired on a Nikon Ti Eclipse fluorescence microscope using the 40x DIC, FITC and TxRed detection channels and processed in Nikons own NIS-Elements software. Infective cysts stain positive for FDA and show a more structured interior and a halo in the DIC channel, while non-infective cysts have lost the ability to activate FDA and their cell membrane is leaky, so they stain positive for PI.

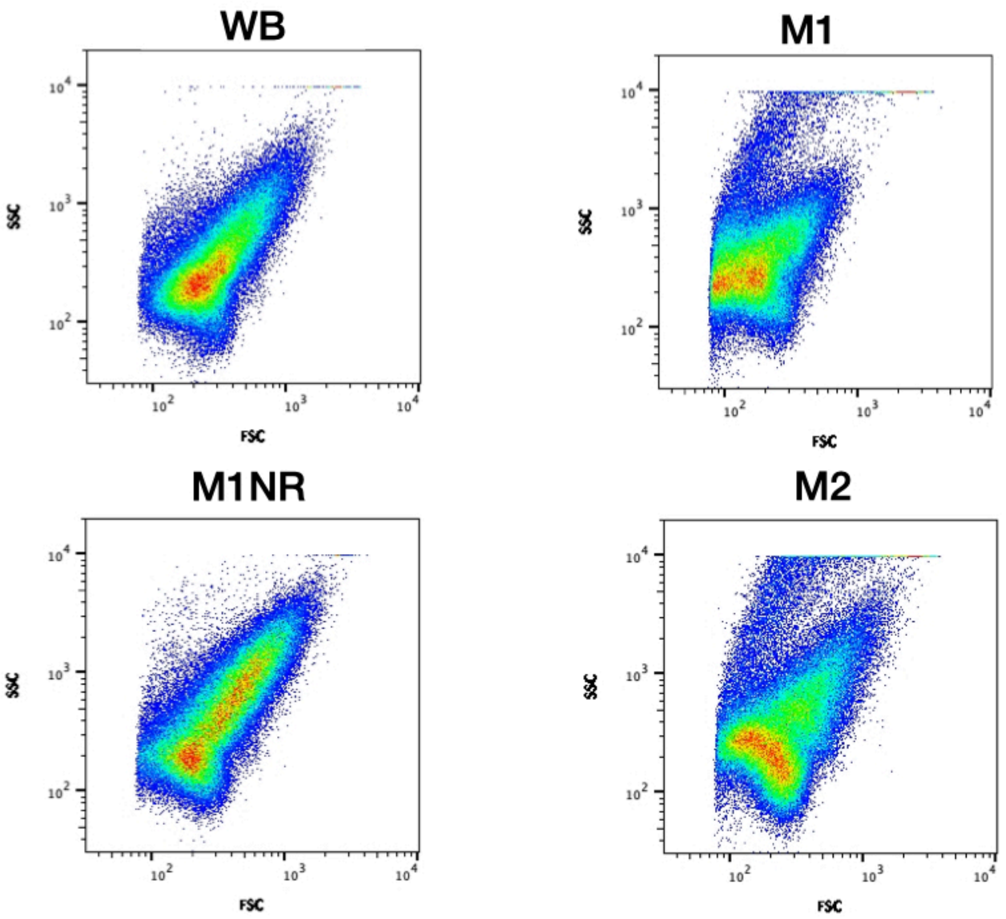

Supplementary Figure 2. Forward and sideward scattering of cysts from lines WB-C6, M1, M1NR and M2. On the x-axis the forward scattering of cysts can be seen, giving a rough estimate of the size of the particles measured, on the y-axis the sideward scattering can be seen, giving a rough estimate of the intracellular complexity of the particles measured. Every dot represents on event. The flow cytometer parameters were set using a WB-C6 sample.

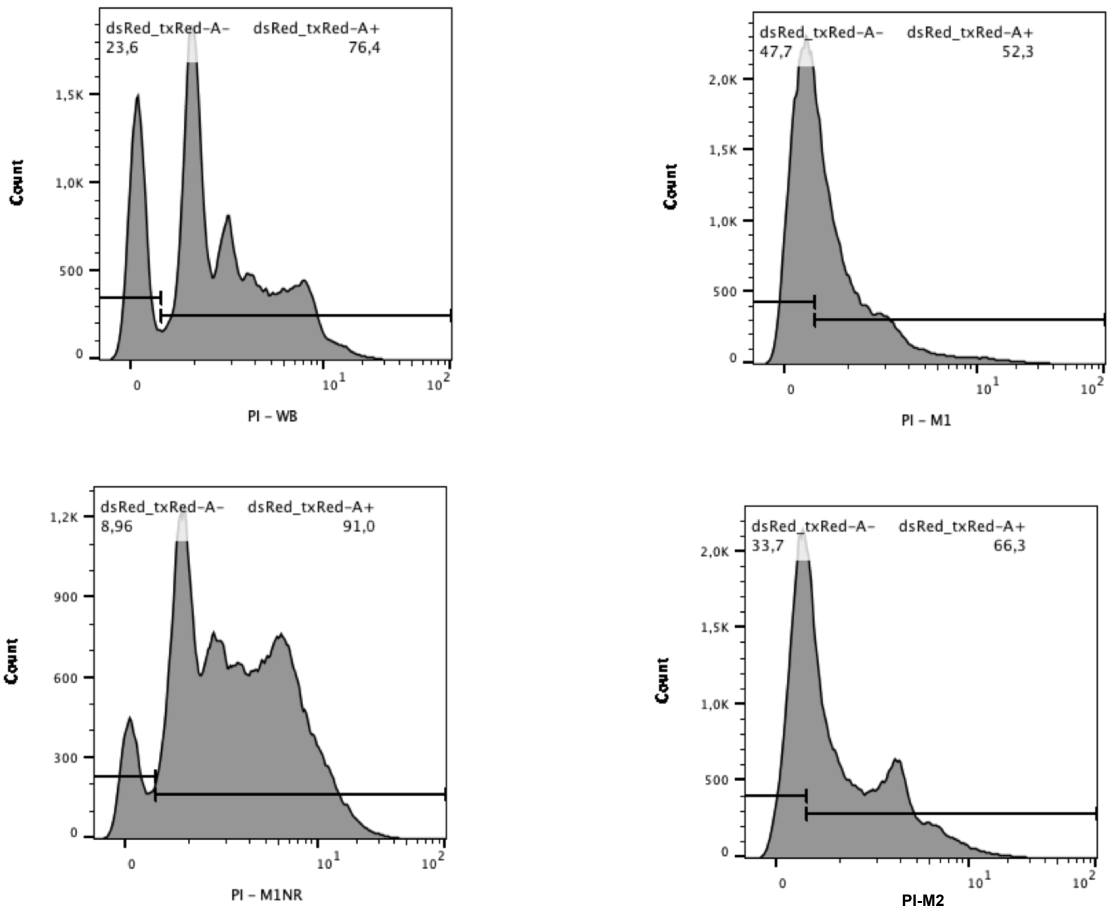

Supplementary Figure 3. Example histograms of PI staining for lines WB-C6, M1, M1NR and M2. On the X-axis the relative strength of fluorescence is shown, while on the y-axis the counts for each relative strength are depicted. The negative/positive separation was created based on the WB-C6 curves; population percentages for the samples depicted here are shown in the top of each plot. Alive cells should not stain for PI at all, since an intact membrane prevents PI from entering the cell. Different PI strengths represent different amounts of DNA.

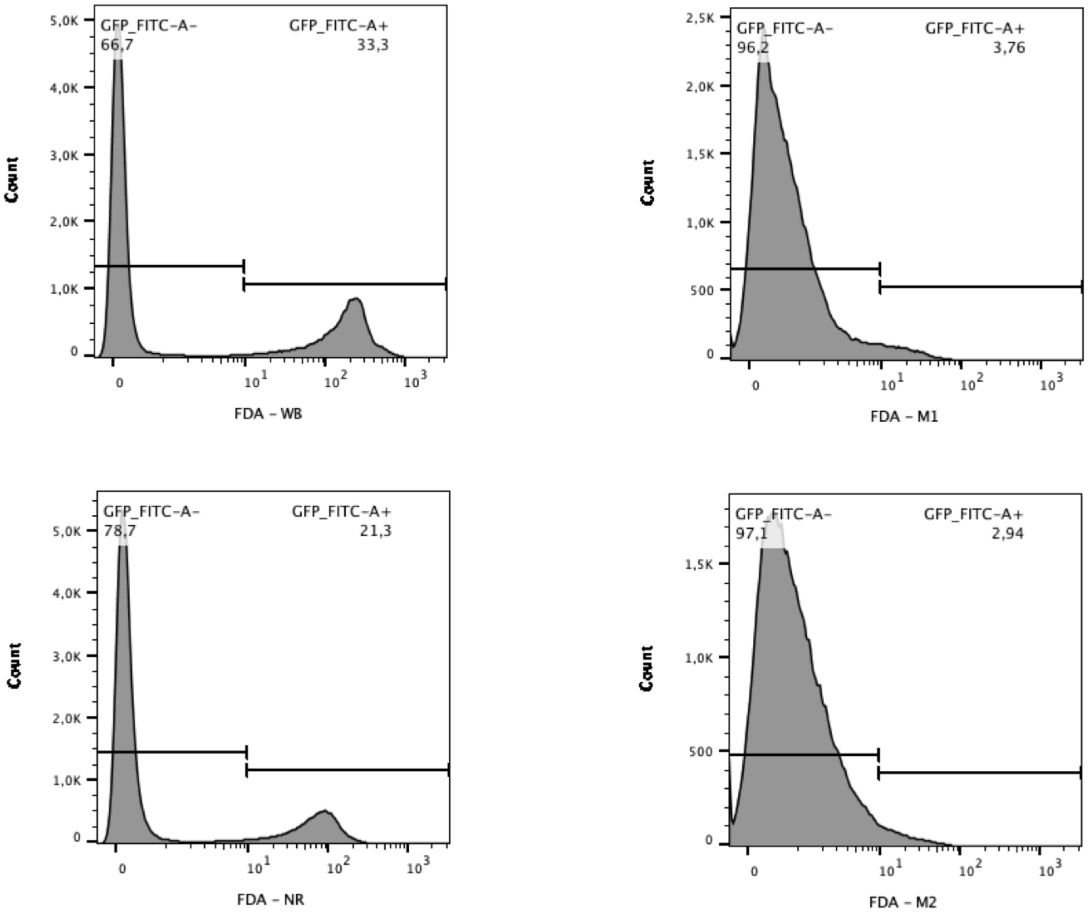

Supplementary Figure 4. Example histograms of FDA staining for lines WB-C6, M1, M1NR and M2. On the X-axis the relative strength of fluorescence is shown, while on the y-axis the counts for each relative strength are depicted. The negative/positive separation was created based on the WB-C6 curves; population percentages for the samples depicted here are shown in the top of each plot. Alive cells will stain green for FDA since they can activate the molecule and retain the active form inside the cell thanks to their cell membrane.

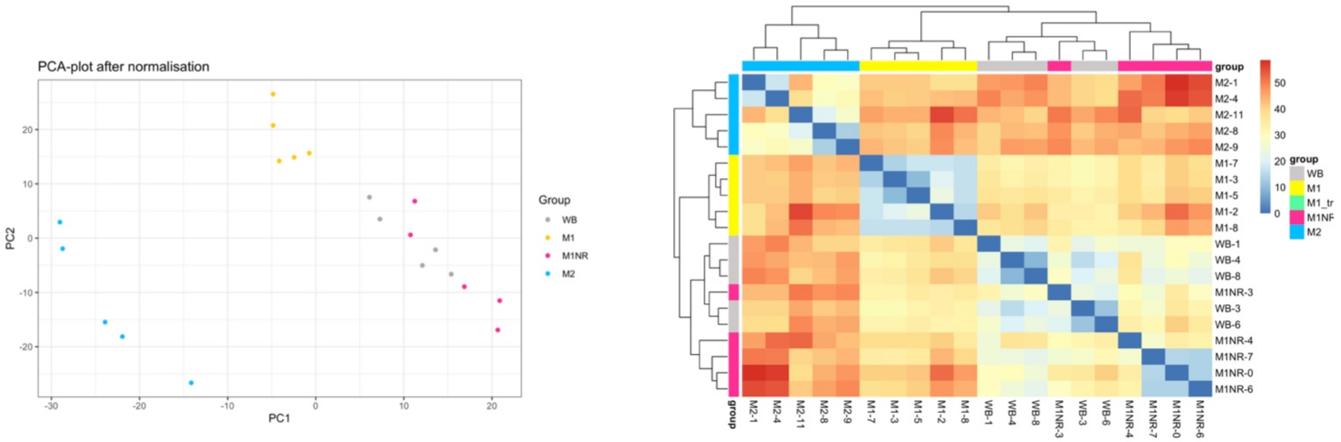

Supplementary Figure 5. Clustering of RNA samples. On the left hand side, a PCA plot can be seen for the samples from WB-C6, M1, M1NR and M2. On the right hand side a clustering heatmap can be seen for the same samples. Closeness to other samples is given on an artificial scale from 0 to 60 with 0 being exactly identical.

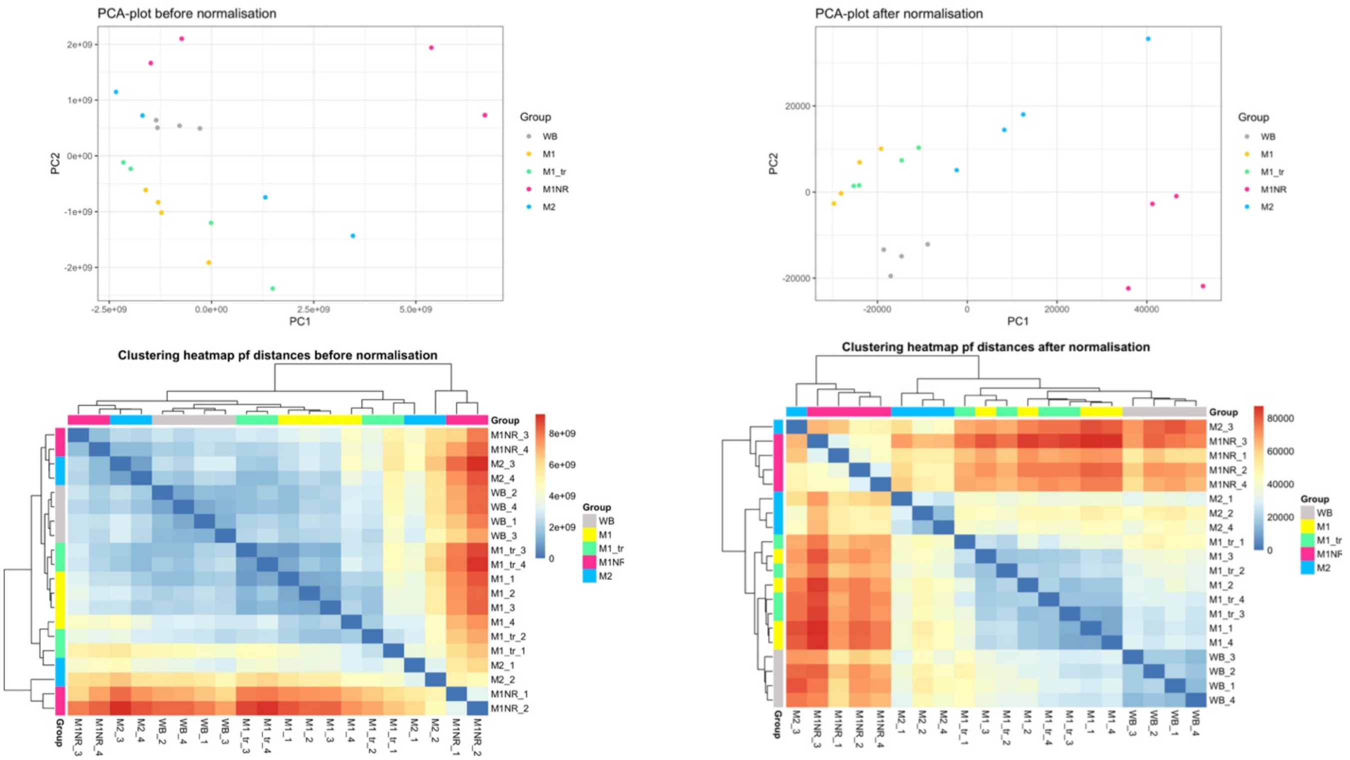

Supplementary Figure 6. Clustering of protein samples. On the left hand side, plots for all samples before normalization with the onboard edgeR function are shown, on the right hand the same plots after normalization. On the upper half of each side a PCA plot can be seen for the samples from WB-C6, M1, M1NR and M2. On the lower half a clustering heatmap can be seen for the same samples. Closeness to other samples is given on an artificial scale from 0 to an upper limit with 0 being exactly identical. As can be seen the sample signal strengths are still very different when compared to the RNA dataset.

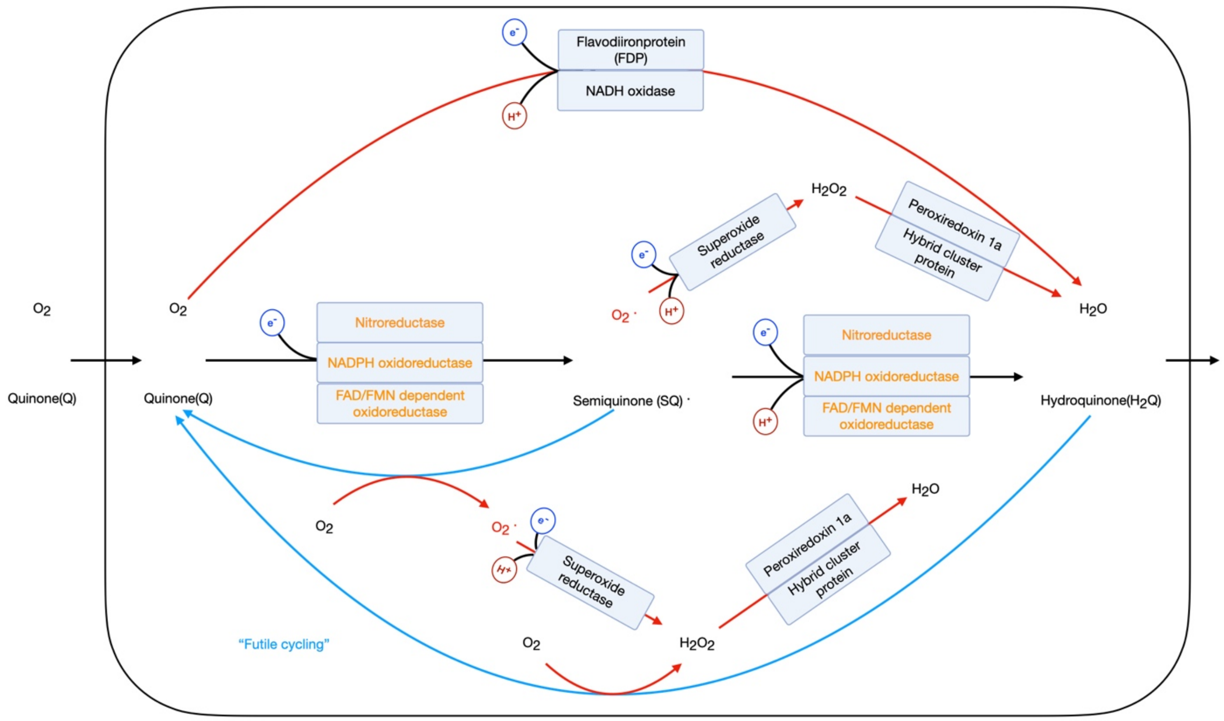

Supplementary Figure 7. Schematic overview of cellular mechanism involving oxygen and quinones in the giardial cell. Metronidazole activating enzymes and molecules are shown in orange text, radicals are shown in red. Red arrows show reduction of oxygen, superoxide and hydrogen peroxide and blue arrows denote futile cycling. Electrons entering the reactions are shown in a blue circle and hydrogen protons in red. Several enzymes on the same reaction arrow show that either of these enzymes can catalyze this reaction.
